# Tissue suction-mediated gene transfer to the beating heart in mice

**DOI:** 10.1371/journal.pone.0228203

**Published:** 2020-02-06

**Authors:** Yota Taniguchi, Natsuko Oyama, Shintaro Fumoto, Hideyuki Kinoshita, Fumiyoshi Yamashita, Kazunori Shimizu, Mitsuru Hashida, Shigeru Kawakami

**Affiliations:** 1 Graduate School of Biomedical Sciences, Nagasaki University, Sakamotomachi, Nagasaki, Japan; 2 Department of Community Medicine Supporting System, Kyoto University Graduate School of Medicine, Kyoto University, Kyoto, Japan; 3 Department of Drug Delivery Research, Graduate School of Pharmaceutical Sciences, Kyoto University, Yoshida-shimoadachi cho, Sakyo-ku, Kyoto, Japan; 4 Graduate School of Engineering, Nagoya University, Furo-cho, Chikusa-ku, Nagoya, Japan; University of Pennsylvania, UNITED STATES

## Abstract

We previously developed an *in vivo* site-specific transfection method using a suction device in mice; namely, a tissue suction-mediated transfection method (tissue suction method). The aim of this study was to apply the tissue suction method for cardiac gene transfer. Naked plasmid DNA (pDNA) was intravenously injected in mice, followed by direct suction on the beating heart by using a suction device made of polydimethylsiloxane. We first examined the effects of suction conditions on transgene expression and toxicity. Subsequently, we analyzed transgene-expressing cells and the transfected region of the heart. We found that heart suction induced transgene expression, and that −75 kPa and −90 kPa of suction achieved high transgene expression. In addition, the inner diameter of the suction device was correlated with transgene expression, but the pressure hold time did not change transgene expression. Although the tissue suction method at −75 kPa induced a transient increase in the serum cardiac toxicity markers at 6 h after transfection, these markers returned to normal at 24 h. The cardiac damage was also analyzed through the measurement of hypertrophic gene expression, but no significant differences were found. In addition, the cardiac function monitored by echocardiography remained normal at 11 days after transfection. Immunohistochemical analysis revealed that CD31-positive endothelial cells co-expressed the ZsGreen1-N1 reporter gene. In conclusion, the tissue suction method can achieve an efficient and safe gene transfer to the beating heart in mice.

## Introduction

Although there have been many efforts to develop pharmacological drugs and surgical devices to combat heart failure, it remains the major cause of death and hospitalization [1]. It is reported that more than 23 million people in the world have heart failure-related diseases. In the past two decades, our knowledge of the molecular pathways associated with heart failure have increased, indicating potential targets for the cure of cardiac disorders [2–4]. As it is difficult to control these signaling pathways by using pharmacological reagents such as small molecule inhibitors, gene therapy has emerged as a possible strategy against heart failure [3, 4]. However, many issues need to be resolved, including transfection efficiency, tissue specificity, toxicity, and immune activity. For example, gene transfer techniques using viral vectors can achieve high transfection efficiency, but often result in off-target gene expression in unintended tissues, such as the liver [5]. In contrast, non-viral vectors such as plasmid DNA (pDNA) have limited immunogenicity, but achieve low transfection efficiency [3, 4]. These problems may affect the clinical outcomes and preclinical results. Thus, organ-specific and safe gene delivery systems are needed for both clinical and experimental use.

Previously, we developed a tissue suction-mediated transfection method (tissue suction method) [6–8]. This is a simple gene delivery method: naked nucleic acids, such as pDNA and siRNA, are injected intravenously, followed by the application of suction pressure on the target organ. Previously, we have demonstrated that this tissue suction method of gene transfer can be applied for transfection of the liver, kidney, heart, and spleen of mice [6]. Moreover, this transfection technique did not cause severe damage when applied to the liver [6, 7] and kidney [8] of mice. Hence, a cardiac suction method should offer a promising approach for the progression of gene functional analysis and clinical gene therapies. The parameters related to the transfection efficiency and toxicity should be optimized to establish a reproducible transfection method. In addition, it is essential to understand the transfected cell types to select suitable genes for the treatment of cardiac dysfunction. However, there have been few studies of the effect of the physical stimuli by suction on the heart.

In the present study, we examined the effect of suction conditions on cardiac transfection using a computer-regulated tissue suction device [7, 8]. Then, the possible cardiac damage induced by suction was investigated through the measurement of hypertrophic gene expression, serum cardiac toxicity markers, and echocardiographic parameters. Moreover, we identified the transfected cell types by using immunostaining.

## Materials and methods

### Fabrication of tissue suction device

Three types of suction devices were fabricated, as reported previously [6] (Table 1). Briefly, precured polydimethylsiloxane (10:1) solution was incubated in the molds at 75°C for 12 h. Thereafter, the cured polydimethylsiloxane was formed into individual devices. Individual devices were linked to a silicone tube with an outer diameter of 2 mm. The tube was used to supply the negative pressure. The device height was 3 mm. The inner and outer diameters of the device were designed as indicated in Table 1. Unless otherwise noted, device I was used in the experiments.

**Table 1:** Suction devices.

Device type	Inner diameter	Outer diameter
I	1.5 mm	3 mm
II	2 mm	3 mm
III	3 mm	5 mm

### Suction pressure-controlled computer system

The suction pressure-controlled computer system was constructed as described previously [7]. Briefly, a vacuum pump generated negative pressure, controlled by a PC with LabVIEW software (National Instrument, Austin, TX) via an electropneumatic regulator (ITV0090; SMC, Tokyo, Japan). The actual suction pressure was detected by using a pressure sensor (Sensez, Tokyo, Japan), and saved in the PC.

### pDNA and mice

The pCMV-Luciferase (pCMV-Luc) used was as constructed previously [9]. pZsGreen1-N1 was purchased from Clontech (Takara Bio, Shiga, Japan). The *Escherichia coli* strain DH5a was used for amplifying pDNA. The quality of pDNA was examined by measuring the ratio of absorbance at 280 nm to that at 260 nm. Five-week-old female ICR mice were purchased from Japan SLC (Shizuoka, Japan). All animal experiments were conducted in accordance with the Guide for the Care and Use of Laboratory Animals, as adopted and promulgated by the United States National Institutes of Health (Bethesda, MD). The study protocol permission numbers 2013–41, 2014–32, and 2014–56 were approved by the Animal Research Committee, Kyoto University, Japan, and 1812251497–2 was approved by the Institutional Animal Care and Use Committee of Nagasaki University, Japan.

### *In vivo* transfection by the tissue suction method

The mice were anesthetized with isoflurane or three types of mixed anesthetic agents (0.75 mg/kg medetomidine, 4.0 mg/kg midazolam, and 5.0 mg/kg butorphanol) and their respiration was maintained artificially at 1 cm^3^ and 100 rpm by using a ventilator (SN-480-7; Sinano-Seisakusho, Tokyo, Japan) after tracheal intubation. When anesthesia and respiration were controlled, the left chest costal between the third rib and fourth rib was cut to give minimal exposure of the left ventricle. Mice were intravenously injected with 200 μL of saline containing 100 μg pDNA, and the ventricle was suctioned immediately by a pressure-controlled suction device. At the end of the surgery, the chest was closed by using suture threads. Sham-operated mice underwent the same procedure, without suction. At the end of studies, all mice were sacrificed by cervical dislocation, or blood removal from vena cava under anesthesia.

### Luciferase assay

Luciferase assays was performed as previously described [9]. The mice heart and the other organs were dissected 6 h after transfection. The organs were stored at −80°C before each assay was performed. The luciferase activities of the organ lysate were determined by using a PicaGene Luminescent Kit (Toyo Ink, Tokyo, Japan) and luminometer (Lumat LB 9507; EG&G Berthold, Bad Wildbad, Germany). Each luciferase activity measurement was normalized to protein content, which was determined by using a Protein Quantification Kit (Dojindo Molecular Technologies, Tokyo, Japan).

### Quantitative real-time RT-PCR

Hypertrophic genes, *α-myosin heavy chain (MyHC)*, *β-MyHC*, *SERCA*, *atrial natriuretic factor (ANF)*, and *B-type natriuretic peptide (BNP)* were measured by using real-time RT-PCR. At 48 h after transfection, the heart was dissected and minced by scissors to pieces smaller than 5 mm. Total mRNA was extracted from the minced organ by using RNeasy Fibrous Tissue Mini Kit (74704; Qiagen, Hilden, Germany). Thereafter, reverse transcription of mRNA and real-time PCR using SYBR Premix Ex Taq (RR039A; Takara Bio) was performed as described previously [10]. The sequences of primers used in the present study are shown in Table 2.

**Table 2:** Primer sequences used for quantitative real time RT PCR.

Name	Forward (5’-3’)	Reverse
*αMyHC*	CCAGTACTTTGCCAGCATTGCAGC	ACACCTATGAAGTACTGGCGCGGC
*βMyHC*	AAGTGAAGAGCCTCCAGAGTCTGC	GGGCTTCACGGGCACCCTTAGAGC
*Serca*	GCATTGCAGTCTGGATCATCAACA	GCCACCATGAACTGGGTCATT
*BNP*	AAGCTGCTGGAGCTGATAAGA	GTTACAGCCCAAACGACTGAC
*ANF*	ACGCCAGCATGGGCTCCTTCTCC	GCTGTTATCTTCGGTACCGGAAG
*GAPDH*	TCTCCTGCGACTTCAACA	GCTGTAGCCGTATTCATTGT

### Evaluation of creatine kinase-muscle/brain (CK-MB) and troponin T2 (TNNT2)

Six or twenty-hour after transfection, the mice were anesthetized, and blood samples were collected from the abdominal vena cava of each mouse. The collected samples were left overnight at 4°C, and centrifuged at 3,000×g for 15 min at 4°C to obtain the serum from the supernatant. Separated serum was stored at −80°C before the assays were performed. Creatine kinase-muscle/brain (CK-MB) and troponin T2 (TNNT2) levels in the serum were determined by using a commercial enzyme-linked immunosorbent assay kit (Wuhan USCN Business, Houston, Texas, USA) in accordance with the recommended procedures.

### Hematoxylin and eosin staining

Twenty four-hour after transfection, mice were sacrificed, and the hearts were removed. Collected mouse tissues were fixed in 4% phosphate-buffered paraformaldehyde and embedded in paraffin. The samples were sliced into 5 μm sections, the paraffin was removed by application in xylene, and the slices were rehydrated in graded alcohol series. The sections were then stained with hematoxylin and eosin (HE). The histology of the heart sections was examined by using a microscope (BZ-X700; Keyence, Osaka, Japan).

### Echocardiographic analysis

Echocardiography was performed at 11 days after transfection as previously described [11] using a Toshiba PowerVision 8000 (Toshiba, Tokyo, Japan) equipped with a 12 MHz imaging transducer.

### Immunohistochemistry

The heart was dissected at 24 h after transfection of pZsGreen1-N1. The frozen sections of the heart were prepared and fixed with acetone for 10 min. Non-specific binding to the sections was blocked by incubation in 5% normal goat serum (G9023, Sigma Aldrich, St. Louis, Missouri, USA) for 30 min at room temperature. Thereafter, the samples were incubated with primary antibodies against CD31 (1:100 dilution, #102401; BioLegend, San Diego, CA, USA) and α-sarcomeric actin (1:100 dilution, ab68167; Abcam, Boston, MA, USA) overnight at 4°C. The samples were then washed with PBS, and reacted with the following secondary antibodies for 1 h at room temperature (1–30°C): Alexa Fluor 647-conjugated antibody against rat IgG (1:200 dilution, 112-605-167; Jackson Immuno Research Laboratories, West Grove, PA); Alexa Fluor 555-conjugated antibody against rabbit IgG (1:500 dilution, #4413; Cell Signaling Technology, Danvers, MA). The stained sections were observed by using confocal laser scanning microscopy (CLSM).

### Statistical analysis

Statistical significance was determined by analysis of variance (ANOVA) for multiple comparisons among different groups, followed by the Tukey-Kramer test. For the comparisons between two independent groups, unpaired *t*-tests were used. All *P* values were two-tailed, and values of *P* < 0.05 were considered to indicate statistical significance.

## Results

### Evaluation of the effects of suction conditions on transgene expression

#### Effects of varying suction pressure

**Fig 1:**
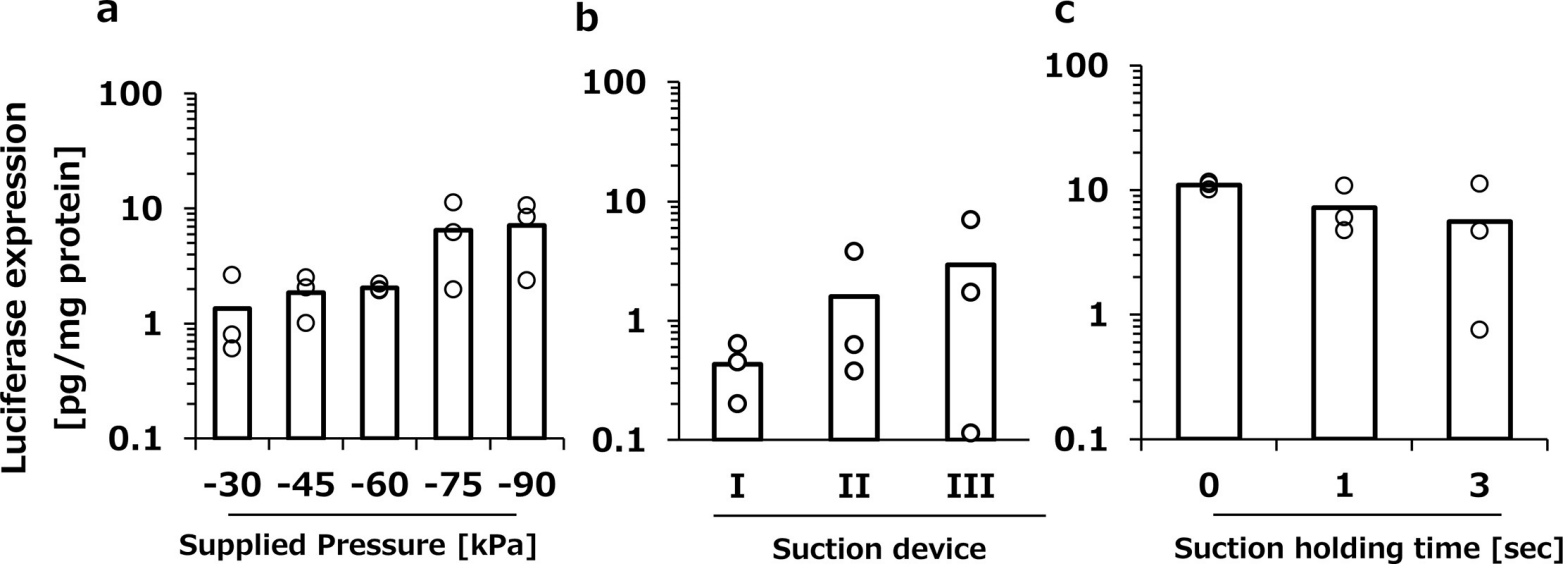
The effect of pressure conditions on transgene expression in the heart. a, Luciferase expression in the heart induced by various degrees of suction pressure. The heart was suctioned at −30, −45, −60, −75, and −85 kPa following intravenous injection of pCMV-Luc solution (100 μg/200 μL/head). b, Luciferase expression in the heart induced by tissue suction with devices with different inner diameters. Suction devices I, II, and III were used for cardiac transfection. c, Luciferase expression in the heart was induced by tissue suction with various pressure hold times. The heart was suctioned at −75 kPa with pressure holding times of 0, 1, and 3 s. Each item represents the pressure supply-hold-release time. a–c, The transgene expression was determined by luciferase assay at 6 h after transfection. Open columns and open dots represent the mean and individual data, respectively (n = 3).

Previously, we developed a suction pressure control system [7, 8]. Our system can regulate the minimum magnitude of the suction pressure and waveform, which comprises pressure supply time, pressure hold time, and pressure release time. First, the pressure supply time, hold time, and release time were set to 1 s, 3 s, and 1 s, respectively, and various negative pressures were applied to the heart. Although there was no significant difference, when the tissue suction was set to −75 kPa and −90 kPa, higher transgene expression tended to be induced in the heart (Fig 1A). Unless otherwise noted, the suction pressure was set to −75 kPa in the subsequent studies.

#### Effects of suction device size

Next, the transfection was performed by using three different sizes of the device (Table 1). In this experiment, pressure supply time, hold time, and release time were set to 1 s, 3 s, and 1 s, respectively, and the minimum pressure was set to −75 kPa. Tissue suction using device III induced the highest transgene expression (Fig 1B). However, the larger sized suction device would have a greater impact on the near organs. Since we would like to focus on the direct effects on the heart function, we used device I having minimum diameter in the subsequent experiments.

#### Effects of pressure hold time

To minimize the duration of suction, the effect of pressure hold time on the transgene expression was investigated (Fig 1C). Pressure hold time was varied from 0 s to 3 s when the negative pressure condition was set to −75 kPa and the pressure supply time and release time were set to 1 s. There were no significant differences in the transgene expression when 0 s, 1 s, and 3 s were used for the pressure hold time (Fig 1C). In the following studies, pressure hold time was set to 1 s to obtain reproducible results.

### Evaluation of the possibility of cardiac injury induced by tissue suction

#### Changes in hypertrophic gene expression and serum cardiac toxicity markers

**Fig 2:**
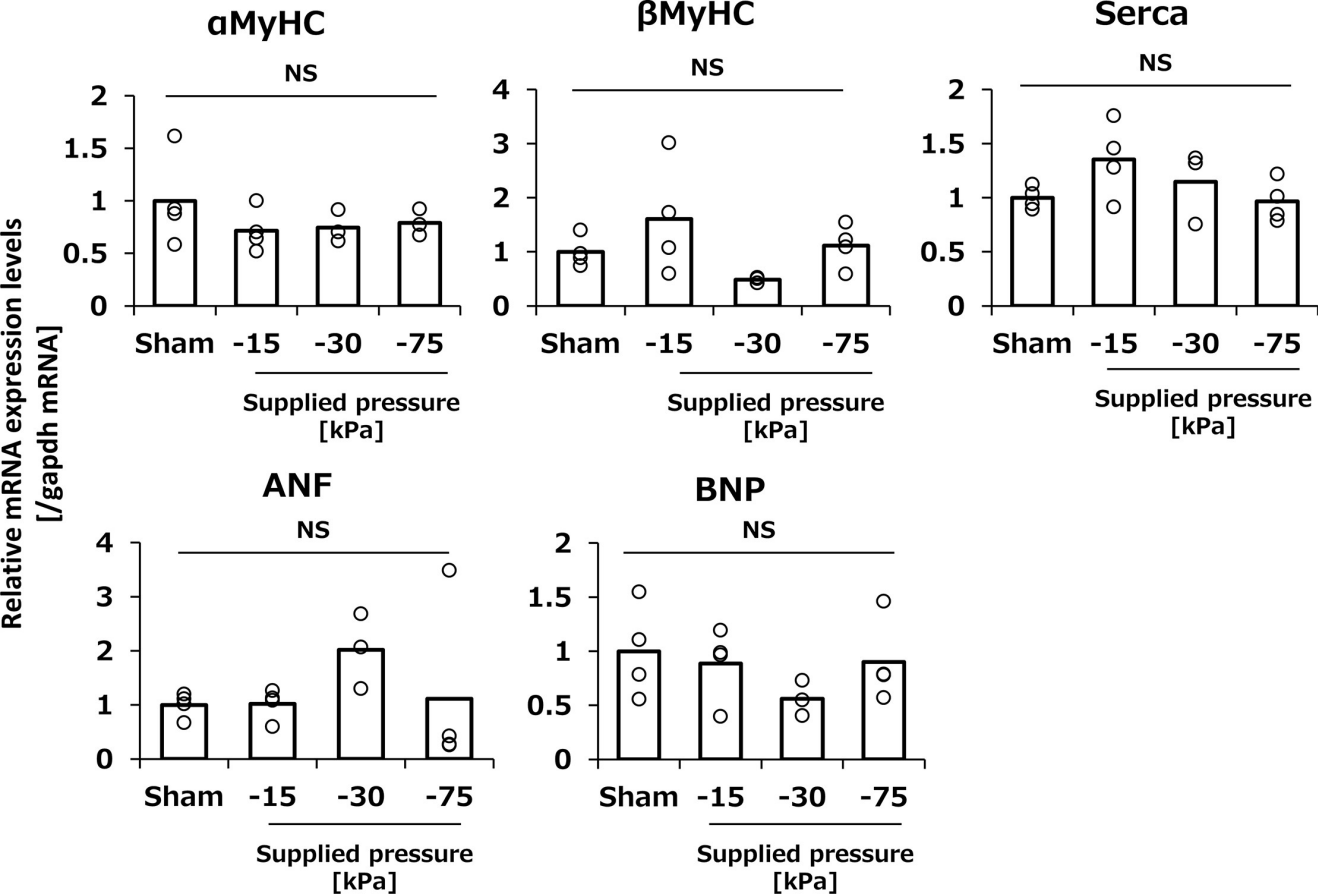
The effect of tissue suction on the cardiac function-related gene expressions. The expression of cardiac function-related genes of the heart after tissue suction. The hearts were dissected at 48 h after transfection. *α-MyHC*, *β-MyHC*, *SERCA*, *ANF*, and *BNP* expression in whole heart tissue was determined by real-time PCR. Open columns and open dots represent the mean and individual data, respectively (n = 3–4). ns: not significant.

**Fig 3:**
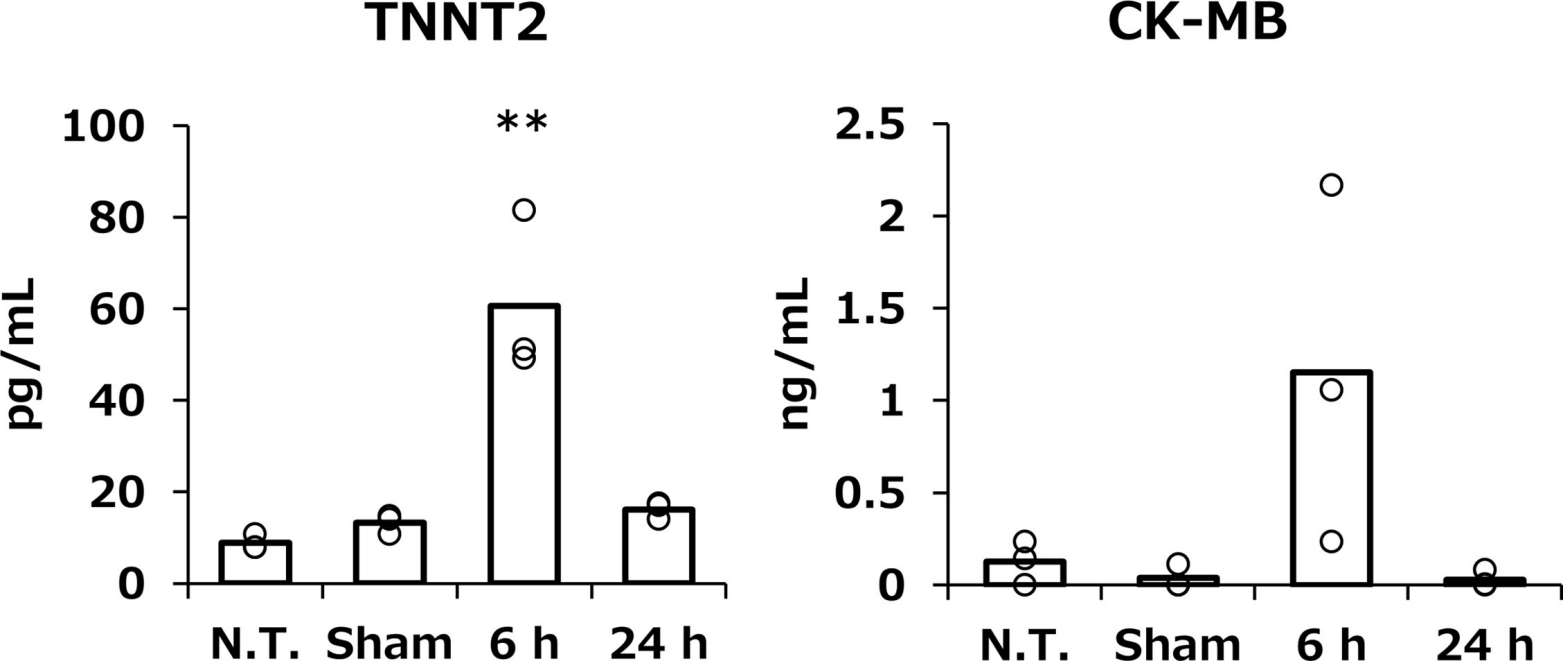
The effect of tissue suction on the serum cardiac toxicity markers. The serum levels of cardiac toxicity markers at 6 and 24 h after pDNA transfection. Serum TNNT2 and CK-MB levels of sham group were measured at 24 h post operation. Serum TNNT2 and CK-MB were determined by using commercially available ELISA kits. Open columns and open dots represent the mean and individual data, respectively (n = 3–4). **p<0.01 compared with all other groups.

Acute cardiac damage, such as myocardial infarction, has been reported to affect hypertrophic gene expression [12]. For this reason, the hypertrophic gene expression levels of *αMyHC*, *βMyHC*, *Serca*, *ANF*, and *BNP* were analyzed 48 h after tissue suction (Fig 2). In this experiment, the pressure degree was varied from set to −15, −30 and −75 kPa. As a result, these hypertrophic genes were hardly affected by tissue suction (Fig 2). Consequently, the two types of serum cardiac toxicity markers, CK-MB and TNNT2, were evaluated followed by a suction set to −75 kPa. Although both serum cardiac injury markers significantly elevated compared to sham group at 6 h after transfection, both reversed normal levels at 24 h (Fig 3).

#### Histological analysis of tissue sections

**Fig 4:**
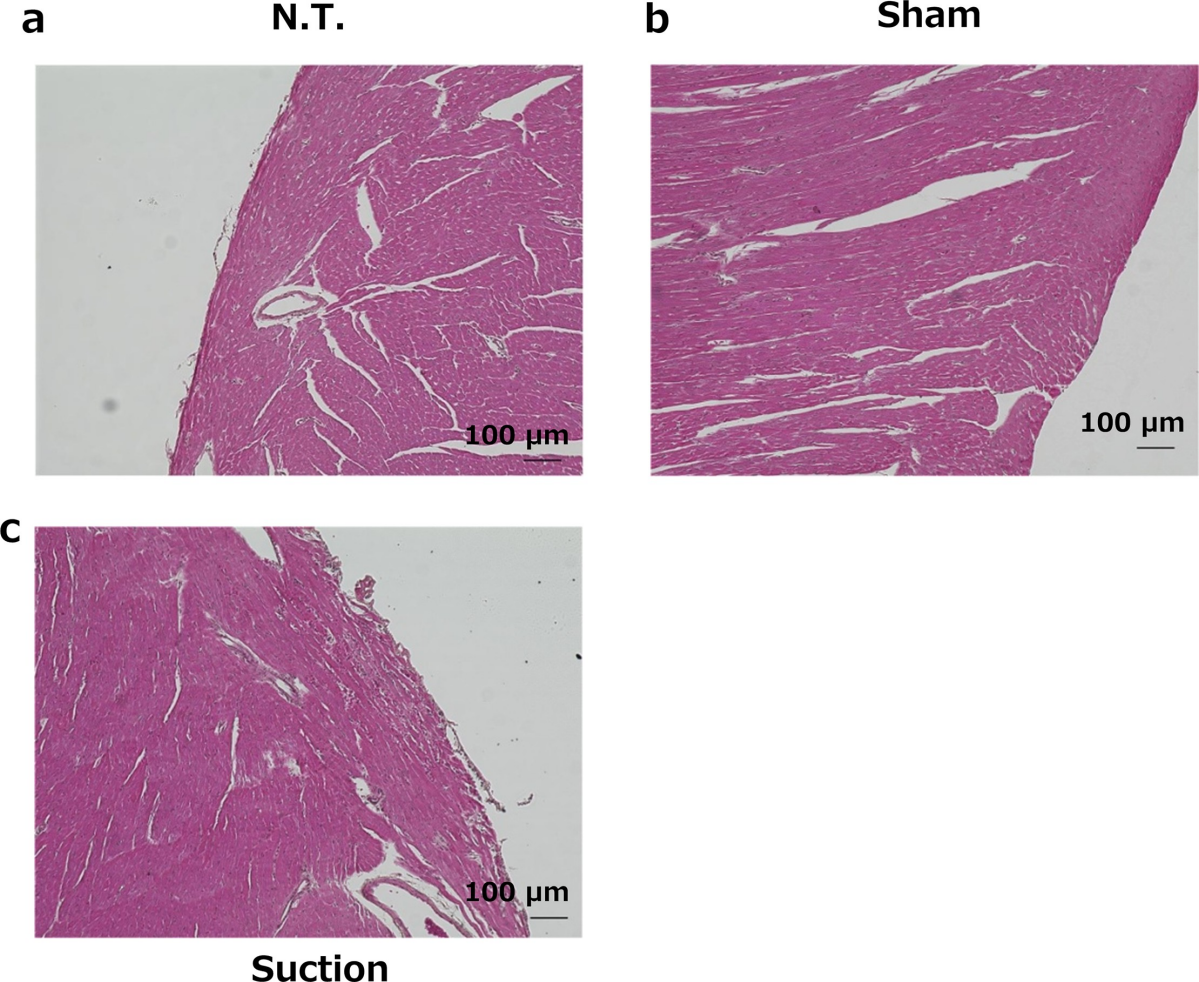
Histological assessment of HE stained cardiac sections. The hearts were dissected at 24 h after transfection. Five micrometer thick sections were stained with HE. a, Normal mouse heart. b, Sham operated mouse heart. c, Suctioned heart (−75 kPa). Scale bars = 100 μm.

Tissue sections of the suctioned heart were stained with HE to reveal histological abnormalities. Suction at −75 kPa did not induce histological abnormalities at 24 h after transfection (Fig 4).

#### Electrocardiographic analysis

Severe cardiac damage, such as myocardial infarction, has been reported to impair cardiac function after 1 week [13]. For this reason, we performed echocardiography at 11 days after transfection to evaluate the effect of tissue suction on cardiac function. There were no significant differences between the groups transfected by cardiac suction and the sham-operated group (Table 3).

**Table 3:** Echocardiographic analysis at 11 days after transfection.

	Sham (n = 3)	Suction (n = 3)	p value
LVDd (mm)	2.1 ± 0.1	2.0 ± 0.1	0.27
LVDs (mm)	0.9 ± 0.1	0.8 ± 0.1	0.57
FS (%)	54 ± 5.5	59 ± 2.5	0.46
EF (%)	90 ± 3.8	93 ± 1.3	0.50
IVS (mm)	0.8 ± 0.03	0.8 ± 0.00	0.42
PW (mm)	0.8 ± 0.06	0.9 ± 0.06	0.29
HR (/min)	680 ± 18	684 ± 12	0.84

Values are presented as the mean ± SEM (n = 3). LVDd, left ventricular end diastolic dimension; LVDs, left ventricular end systolic dimension; FS, fractional shortening; EF, ejection fraction; IVS, interventricular septal thickness; PW, posterior wall thickness; HR, heart rate. Unpaired *t*-tests were used for the comparisons between two groups.

### Distribution of transgene expression

#### Selective gene transfer to the heart

**Fig 5:**
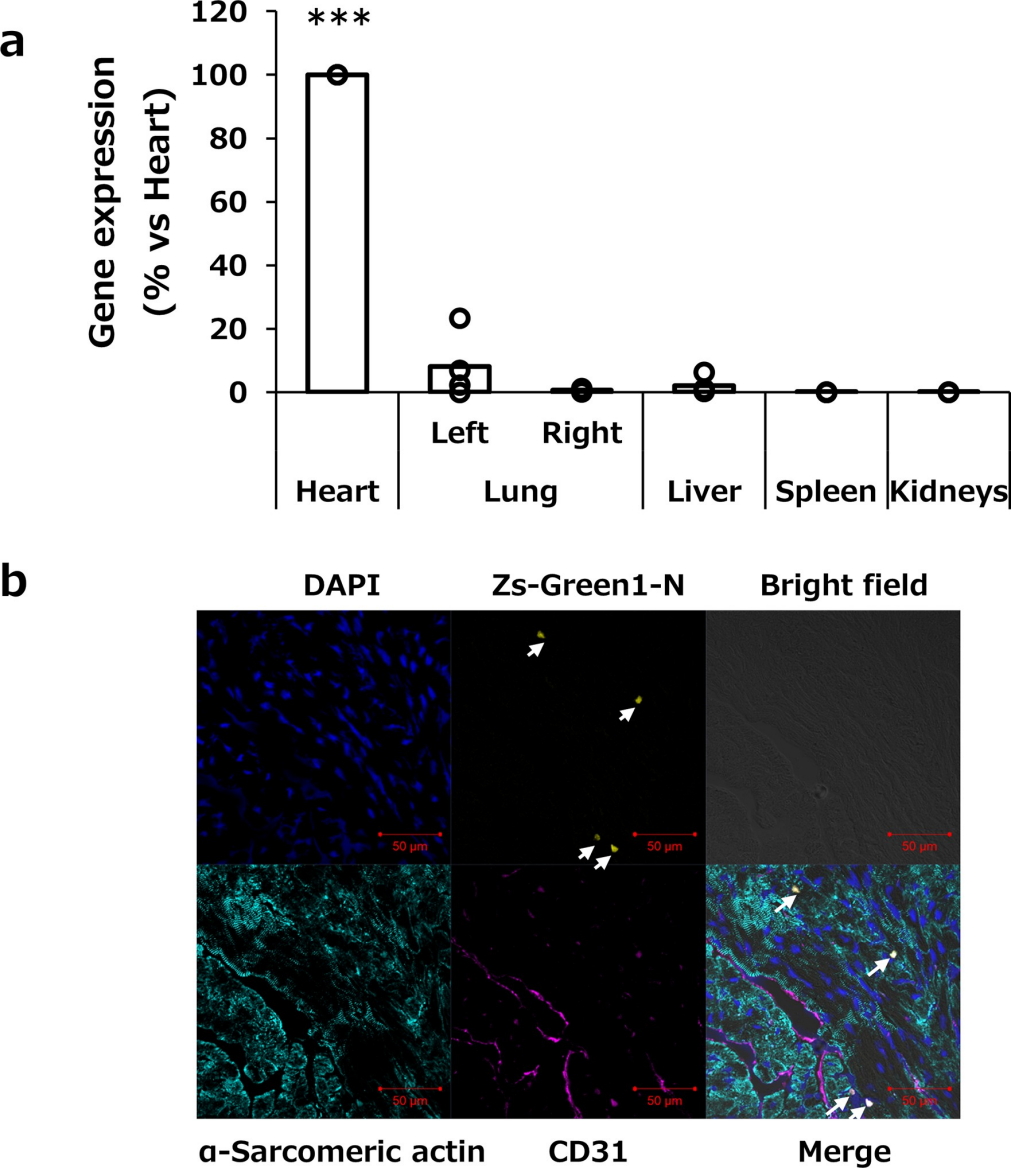
Distribution of transgene expression and identification of transfected cell types. a, Selective gene transfer to the heart by the tissue suction method. The heart was suctioned at −75 kPa. At 6 h after transfection, various tissues (the heart, left and right lungs, liver, spleen, and kidneys) were collected. Transgene expression in each organ was determined by luciferase assay and normalized to the expression of cardiac tissue. Open columns and open dots represent the mean and individual data, respectively (n = 3). ***p<0.001 compared with all other groups. b, Analysis of transgene-expressing cell types by immunostaining of endothelial cells and cardiac muscle cell markers. The pZsGreen1-N1 was transfected to the heart by the tissue suction method. The heart was suctioned at −75 kPa and then collected at 24 h after transfection. Finally, 10 μm-thick frozen sections were stained with anti-CD31 and α-sarcomeric actin antibodies and DAPI. The stained sections were observed by using CLSM. Magnification ×40. Blue: nucleus; yellow: ZsGreen1-N; cyan: α-sarcomeric actin-positive cardiac muscle cells; magenta: CD31-positive endothelial cells. Scale bars = 50 μm. White arrows indicate ZsGreen1-expressing cells.

To evaluate the organ selectivity of the transgene expression, luciferase expression in the heart, right and left lungs, liver, spleen, and kidneys were analyzed at 6 h after transfection. As shown in Fig 5A, the transgene expression was mainly observed in the suctioned heart and partially at the left lung, up to 23% compared with the heart.

#### Identification of transgene-expressing cells

To clarify the transfected cell types, immunohistochemical analysis was performed 24 h after transfection of the ZsGreen1-N1 gene. Cardiomyocytes and vascular endothelial cells were stained with an anti α-sarcomeric actin antibody and an anti-CD31 antibody, respectively. ZsGreen1-N1 expression was mainly co-localized with CD31-positive endothelial cells (Fig 5B).

## Discussion

The body of knowledge on cardiac regeneration and healing pathways has rapidly evolved recently, and reliable methods are needed to verify the therapeutic targets in experimental and clinical studies. Several cardiac gene transfer techniques have been developed. They are broadly divided into viral and non-viral vector-based methods [3]. Viral vectors such as adeno-associated virus (AAV) is often used to transduce therapeutic gene to the heart because of the long-term expression [3, 4]. AAV has a heart selectivity for gene transfection after intravenous administration [5, 14]. Naked non-viral vectors are relatively safe in terms of the immune responses; however, they generally result in poor transgene expression. To overcome these problems, many innovative approaches have been developed. The ultrasound-mediated transfection method has been applied to improve the transfection efficiency of non-viral vectors for the treatment of heart failure in the rodent model [15, 16]. Therefore, we hypothesized that heart selective and enhanced gene expression by naked pDNA would be achieved by the application of the tissue suction method to the beating heart of mice. The simple procedure of the tissue suction method may have an advantage in clinical and preclinical studies. In the present study, we applied the tissue suction method to cardiac gene delivery and this is the first report to clarify the characteristics of the tissue suction-mediated gene transfer in the beating heart in mice. The optimal transgene expression was obtained by using a pressure controlled-suction device set at −75 kPa and this application of optimal suction to the beating heart did not induce severe cardiac injury. Transgene expression induced by tissue suction method reached approximately 10 pg/mg protein in the mouse heart. Fujii H, et al, demonstrated that 4 pg /mg protein of stem cell growth factor protein improved cardiac function in the rodent model [15, 16]. Although the kinetics of transfected protein should be further investigated, transfection efficiency of tissue suction method would be applicable for the treatment of heart disease. We also found that the tissue suction method could target the endothelial cells of the cardiac capillaries.

The expression of cardiac hypertrophy-related genes, *α-MyHC*, *β-MyHC*, *SERCA*, *ANF*, and *BNP*, was not affected by the characteristics of the tissue suction method (Fig 2). We also evaluated the serum levels of two cardiac toxicity markers, TNNT2 and CK-MB. CK-MB is a cardiac specific enzyme present in the myocardial cytoplasm, and TNNT2 is also a cardiac-specific protein mostly binding to the myofibrils and partially existing in the cytoplasm freely [17]. These protein markers are released from the injured myocardium and can be used as indicators of myocardial tissue damage. Although heart suction induced levels of circulating CK-MB and TNNT2 levels at 6 h, the levels were restored to normal at 24 h (Fig 3). Moreover, HE sections and echocardiography revealed that the tissue suction method did not induce severe damage in the heart in terms of cardiac morphology and functions (Fig 4, Table 3). Ultrasound-mediated gene transfer to the heart that can form in pores in the cellular membranes also caused a troponin I transient peak [16]. Although the mechanism of the tissue suction method has not been fully clarified, the holes are expected to be transiently formed on the cell membranes because the tissue suction method can transfer pDNA into the suctioned site. Therefore, the transient peaks of the component myocardial proteins in the serum may reflect the holes in the membranes of cardiomyocytes induced by physical stimuli. In summary, the present study demonstrate that tissue suction method did not induce severe dysfunction in the mouse heart. Unfortunately, we did not take electro-cardiogram. The possibility of arrhythmias induced by tissue suction method should be investigated in the future, especially in the context of application on the clinical settings.

To identify the transfected cell types, we performed immunohistochemical analysis after the transfection of ZsGreen1-N1. In the heart transfected by the suction method, transgene expression was observed in the CD31-positive endothelial cells but not in cardiomyocytes (Fig 5B); however, when the suction method was applied to the kidney, transgene expression was observed in the pericytes but not in the endothelial cells of the kidney [10]. The penetration of macromolecules is prevented by a vascular wall composed of tight junctions, adhesion junctions, and basement membranes [18, 19]. One of the major differences between the kidneys and the heart lies in the endothelial structure. The microvascular endothelium of the kidneys, peritubular capillaries, and glomerular capillaries is fenestrated and transports materials [20]. In contrast, continuous capillaries are found in the heart endothelium [21]. Moreover, it was reported that hydrodynamic injection enlarged endothelium fenestrate, which resulted in gene transfer to hepatocytes [22–24]. Consequently, pDNA may pass through the fenestration in the liver and kidneys, but could not penetrate the blood vessels in the heart because of the continuous nature of the endothelial cells. However, there is little information about the effect of tissue suction on pDNA distribution. A hypothesis about the conditions of a cell membrane and the distribution of pDNA is needed for future analysis.

The present study focused on the characterization of the tissue suction method. Two-dimensional analysis revealed that the tissue suction method could target endothelial cells in the heart tissue (Fig 5B). Deng *et al*. have demonstrated that intercellular-adhesion-molecule-1-targeted microbubbles had successfully delivered the angiopoetin-1 gene to inflammatory endothelial cells and improved cardiac function of mice with myocardial infarction [25]. More recently, Hao *et al*. reported that CXCR7, which is a chemokine receptor for CXCL12, was a key regulator for angiogenesis in the endothelium [26]. The information about transfected cells may be of value for future studies of the relationship between the therapeutic gene expression and their pharmacological effects.

For safe gene therapy, expression of a therapeutic gene should be localized in the target organ. We previously reported that kidney or liver suction achieved suction site-specific transgene expression [6, 8]. However, in the case of cardiac transfection, the tissue suction method induced the transgene expression not only in the heart, but also partially in the left lung (Fig 5A). In general, the availability of the naked pDNA to lung cells is very low after intravenous administration [6]. Consistent with the past observations, transgene expression was not observed in the right lung. When these were taken into consideration, we speculated that tissue deformation induced by the device application resulted in gene expression in the left lung. We demonstrated that the larger device was more effective for gene transfection (Fig 1B) because of its large suctioned area. It would there be important to optimize the size of the suction device for each animal to obtain selective and efficient gene transfer to the heart.

Functional RNA molecules, such as micro RNA, small interfering RNA (siRNA), and antisense oligonucleotides, are a promising research tool in functional genomics, and are considered as therapeutic pharmaceuticals [27, 28]. In the present study, we showed that transgene expression might be occurred in the endothelial cells of the mouse heart (Fig 5). We have previously reported a tissue suction method that could deliver siRNA to the mouse liver [6]. Although further studies about the transfection efficiency and the ratio and types of the delivered cells is needed, the tissue suction method may be useful for the analysis of a gene function using oligonucleotides in the mouse heart and may be adaptable to the clinical therapy of heart diseases by oligonucleotide-based drugs.

In the present study, we have investigated the transgene expression only at 6 hr after transfection because the aim of this study is to evaluate the effect of transfection parameter on a transfection efficiency and cardiac functions. In a translational research, the kinetics of a transfected protein should be analyzed. We and other group have previously reported that pDNA removed CG motif (CpG free pDNA) could be used for sustained gene expression [10, 29–30]. We would like to evaluate the kinetics of a therapeutic protein in the future study using CpG free pDNA. In conclusion, the present study showed the characteristics of transfection by the tissue suction method to the beating heart of the mouse. We demonstrated that tissue suction by the pressure-controlled device was a safe, reproducible, and feasible transfection technique. The polydimethylsiloxane (PDMS)-based suction device used in the present study can be mounted on a microscopy [31]. Recently, minimally invasive thoracic surgery techniques have been developed [32]. Although there are several obstacles to be overcome, such as basic studies on animal models and bridging studies from animals to humans, the tissue suction method may provide an option for the treatment of heart failure in clinical settings in combination with novel surgery techniques.

## References

[1] Lund LH, Savarese G. Global Public Health Burden of Heart Failure. Cardiac Failure Review. 2017;03(01). 10.15420/cfr.2016:25:2 28785469 PMC5494150

[2] Schirone L, Forte M, Palmerio S, Yee D, Nocella C, Angelini F, et al A Review of the Molecular Mechanisms Underlying the Development and Progression of Cardiac Remodeling. Oxid Med Cell Longev. 2017;2017:3920195 Epub 2017/07/29. 10.1155/2017/3920195 28751931 PMC5511646

[3] Hulot JS, Ishikawa K, Hajjar RJ. Gene therapy for the treatment of heart failure: promise postponed. Eur Heart J. 2016;37(21):1651–8. Epub 2016/02/29. 10.1093/eurheartj/ehw019 26922809 PMC4887702

[4] Rincon MY, VandenDriessche T, Chuah MK. Gene therapy for cardiovascular disease: advances in vector development, targeting, and delivery for clinical translation. Cardiovasc Res. 2015;108(1):4–20. Epub 2015/08/05. 10.1093/cvr/cvv205 26239654 PMC4571836

[5] Prasad KM, Smith RS, Xu Y, French BA. A single direct injection into the left ventricular wall of an adeno-associated virus 9 (AAV9) vector expressing extracellular superoxide dismutase from the cardiac troponin-T promoter protects mice against myocardial infarction. J Gene Med. 2011;13(6):333–41. Epub 2011/06/16. 10.1002/jgm.1576 21674736 PMC3984922

[6] Shimizu K, Kawakami S, Hayashi K, Kinoshita H, Kuwahara K, Nakao K, et al In vivo site-specific transfection of naked plasmid DNA and siRNAs in mice by using a tissue suction device. PLoS One. 2012;7(7):e41319 Epub 2012/07/31. 10.1371/journal.pone.0041319 22844458 PMC3402481

[7] Shimizu K, Zhang G, Kawakami S, Taniguchi Y, Hayashi K, Hashida M, et al Liver Suction-Mediated Transfection in Mice Using a Pressure-Controlled Computer System. Biological and Pharmaceutical Bulletin. 2014;37(4):569–75. 10.1248/bpb.b13-00776 24818253

[8] Taniguchi Y, Kawakami S, Fuchigami Y, Oyama N, Yamashita F, Konishi S, et al Optimization of renal transfection using a renal suction-mediated transfection method in mice. J Drug Target. 2016;24(5):450–6. Epub 2015/09/24. 10.3109/1061186X.2015.1087526 . 26390999

[9] Kawakami S, Fumoto S, Nishikawa M, Yamashita F, Hashida M. In vivo gene delivery to the liver using novel galactosylated cationic liposomes. Pharm Res. 2000;17(3):306–13. Epub 2000/05/09. 10.1023/a:1007501122611 . 10801219

[10] Oyama N, Fuchigami Y, Fumoto S, Sato M, Hagimori M, Shimizu K, et al Characterization of transgene expression and pDNA distribution of the suctioned kidney in mice. Drug Deliv. 2017;24(1):906–17. Epub 2017/06/07. 10.1080/10717544.2017.1333171 . 28585867 PMC8241128

[11] Kuwahara K, Saito Y, Takano M, Arai Y, Yasuno S, Nakagawa Y, et al NRSF regulates the fetal cardiac gene program and maintains normal cardiac structure and function. EMBO J. 2003;22(23):6310–21. Epub 2003/11/25. 10.1093/emboj/cdg601 14633990 PMC291842

[12] Port JD, Walker LA, Polk J, Nunley K, Buttrick PM, Sucharov CC. Temporal expression of miRNAs and mRNAs in a mouse model of myocardial infarction. Physiol Genomics. 2011;43(19):1087–95. Epub 2011/07/21. 10.1152/physiolgenomics.00074.2011 21771878 PMC3217325

[13] Zhang H, Chen X, Gao E, MacDonnell SM, Wang W, Kolpakov M, et al Increasing cardiac contractility after myocardial infarction exacerbates cardiac injury and pump dysfunction. Circ Res. 2010;107(6):800–9. Epub 2010/07/31. 10.1161/CIRCRESAHA.110.219220 20671241 PMC3021375

[14] Piras BA, O'Connor DM, French BA. Systemic delivery of shRNA by AAV9 provides highly efficient knockdown of ubiquitously expressed GFP in mouse heart, but not liver. PLoS One. 2013 9 24;8(9):e75894 Epub 2013/08/17. 10.1371/journal.pone.0075894 ; PMCID: PMC3782464. 24086659 PMC3782464

[15] Fujii H, Sun Z, Li SH, Wu J, Fazel S, Weisel RD, et al Ultrasound-targeted gene delivery induces angiogenesis after a myocardial infarction in mice. JACC Cardiovasc Imaging. 2009;2(7):869–79. Epub 2009/07/18. 10.1016/j.jcmg.2009.04.008 . 19608138

[16] Fujii H, Li SH, Wu J, Miyagi Y, Yau TM, Rakowski H, et al Repeated and targeted transfer of angiogenic plasmids into the infarcted rat heart via ultrasound targeted microbubble destruction enhances cardiac repair. Eur Heart J. 2011;32(16):2075–84. Epub 2011/01/05. 10.1093/eurheartj/ehq475 . 21196445

[17] Lund M, French JK, Johnson RN, Williams BF, White HD. Serum troponins T and I after elective cardioversion. Eur Heart J. 2000;21(3):245–53. Epub 2000/01/20. 10.1053/euhj.1999.1745 . 10639307

[18] Hu G, Place AT, Minshall RD. Regulation of endothelial permeability by Src kinase signaling: vascular leakage versus transcellular transport of drugs and macromolecules. Chem Biol Interact. 2008;171(2):177–89. Epub 2007/09/28. 10.1016/j.cbi.2007.08.006 17897637 PMC3001132

[19] Lampugnani MG, Caveda L, Breviario F, Del Maschio A, Dejana E. Endothelial cell-to-cell junctions. Structural characteristics and functional role in the regulation of vascular permeability and leukocyte extravasation. Baillieres Clin Haematol. 1993;6(3):539–58. Epub 1993/09/01. 10.1016/s0950-3536(05)80187-8 . 8025343

[20] Jourde-Chiche N, Fakhouri F, Dou L, Bellien J, Burtey S, Frimat M, et al Endothelium structure and function in kidney health and disease. Nat Rev Nephrol. 2019;15(2):87–108. Epub 2019/01/05. 10.1038/s41581-018-0098-z . 30607032

[21] Okada H, Takemura G, Suzuki K, Oda K, Takada C, Hotta Y, et al Three-dimensional ultrastructure of capillary endothelial glycocalyx under normal and experimental endotoxemic conditions. Crit Care. 2017;21(1):261 Epub 2017/10/24. 10.1186/s13054-017-1841-8 29058634 PMC5651619

[22] Zhang G, Gao X, Song YK, Vollmer R, Stolz DB, Gasiorowski JZ, et al Hydroporation as the mechanism of hydrodynamic delivery. Gene Ther. 2004;11(8):675–82. Epub 2004/01/16. 10.1038/sj.gt.3302210 14724673 PMC4412368

[23] Suda T, Gao X, Stolz DB, Liu D. Structural impact of hydrodynamic injection on mouse liver. Gene Ther. 2007;14(2):129–37. Epub 2006/09/22. 10.1038/sj.gt.3302865 . 16988719

[24] Bonamassa B, Hai L, Liu D. Hydrodynamic gene delivery and its applications in pharmaceutical research. Pharm Res. 2011;28(4):694–701. Epub 2010/12/31. 10.1007/s11095-010-0338-9 21191634 PMC3064722

[25] Deng Q, Hu B, Cao S, Song HN, Chen JL, Zhou Q. Improving the efficacy of therapeutic angiogenesis by UTMD-mediated Ang-1 gene delivery to the infarcted myocardium. Int J Mol Med. 2015;36(2):335–44. Epub 2015/06/03. 10.3892/ijmm.2015.2226 26035181 PMC4501666

[26] Hao H, Hu S, Chen H, Bu D, Zhu L, Xu C, et al Loss of Endothelial CXCR7 Impairs Vascular Homeostasis and Cardiac Remodeling After Myocardial Infarction: Implications for Cardiovascular Drug Discovery. Circulation. 2017;135(13):1253–64. Epub 2017/02/06. 10.1161/CIRCULATIONAHA.116.023027 . 28154007

[27] Lucas T, Bonauer A, Dimmeler S. RNA Therapeutics in Cardiovascular Disease. Circ Res. 2018;123(2):205–20. Epub 2018/07/07. 10.1161/CIRCRESAHA.117.311311 . 29976688

[28] Chen C, Yang Z, Tang X. Chemical modifications of nucleic acid drugs and their delivery systems for gene-based therapy. Med Res Rev. 2018;38(3):829–69. Epub 2018/01/10. 10.1002/med.21479 . 29315675

[29] Mejia LC, Rossman AY, Castlebury LA, White JF Jr. New species, phylogeny, host-associations and geographic distribution of genus Cryptosporella (Gnomoniaceae, Diaporthales). Mycologia. 2011;103(2):379–99. Epub 2011/03/19. 10.3852/10-134 . 21415292

[30] Pringle IA, Hyde SC, Connolly MM, Lawton AE, Xu B, Nunez-Alonso G, et al CpG-free plasmid expression cassettes for cystic fibrosis gene therapy. Biomaterials. 2012;33(28):6833–42. Epub 2012/06/26. 10.1016/j.biomaterials.2012.06.009 . 22727465

[31] Konishi S, Horie T, Kurumi Y, Tani T. Reliable Positioning of Micro Device for Medical Diagnosis and Operation on Pulsating Targets by Pneumatic Suction Device. Journal of Japan Society of Computer Aided Surgery. 2009;11(2):59–64. 10.5759/jscas.11.59

[32] Siu ICH, Li Z, Ng CSH. Latest technology in minimally invasive thoracic surgery. Ann Transl Med. 2019;7(2):35 Epub 2019/03/12. 10.21037/atm.2018.12.47 30854388 PMC6381267

